# Application of family whole-exome sequencing for prenatal diagnosis—an analysis of 357 cases

**DOI:** 10.3389/fmed.2025.1529894

**Published:** 2025-08-04

**Authors:** Yijun Ge, Huizhen Yuan, Yao Yu, Junfang Xiao, Danping Liu, Yongbao Peng, Ying Liu, Shuhui Huang, Bicheng Yang, Yongyi Zou, Yanqiu Liu

**Affiliations:** ^1^Jiangxi Maternal and Child Health Hospital, Nanchang, China; ^2^Gongqing Institute of Science and Technology, Gongqing, China; ^3^Shangrao Maternal and Child Health Hospital, Shangrao, China

**Keywords:** whole exome sequencing, prenatal diagnosis, structural abnormalities, genetics, ultrasound technology

## Abstract

**Objective:**

Translation of fertility risks through whole-exome sequencing of family lines to identify variants that explain patient’s clinical phenotypes.

**Methods:**

1. Using techniques such as amniotic fluid, chorionic villus, or umbilical cord blood sampling, intact fetal cells were extracted for cell culture and subsequently analyzed using chromosomal karyotyping and chromosomal microarray techniques. 2. With fully informed consent, fetuses and their parents whose genetic etiology could not be detected by karyotyping combined with chromosomal microarray technology had their cellular DNA subjected to whole-exome sequencing of the pedigree. 3. Pathogenic variants were screened in combination with fetal ultrasound phenotyping and ACMG variant rating guidelines for variant interpretation, followed by inviting multidisciplinary experts to conduct an in-depth analysis and indicate fetal-related ultrasound abnormalities. 4. Genetic counseling is assisted based on the results.

**Results:**

1. Of the 357 fetuses included in the study, 33 (33/357, 9.24%) had a successful genetic etiology identified through family-wide exome sequencing combined with ultrasound phenotyping. 2. The results showed that skeletal anomalies were the most frequent, accounting for 15 cases (15/33, 45.45%), followed by multiple malformations in 7 cases (7/33, 21.21%), renal anomalies in 3 cases (3/33, 9.09%), soft index anomalies in 2 cases (2/33, 6.06%), neurological anomalies in 2 cases (2/33, 6.06%), cleft lip and palate in 1 case (1/33, 3.03%), cardiac abnormality in 1 case (1/33, 3.03%), hydatidiform mole in 1 case (1/33, 3.03%), and cataract in 1 case (1/33, 3.03%). 3. During whole-exome sequencing, three previously unreported variant sites were identified: *MSX2* (NM_002449.4: c.423_427dupCAATC, p.Arg143Profs*39), *EVC* (NM_153717.2: c.130delC, p.Leu44Phefs*72), and *RYR1* (NM_000540.2: c.14129 + 1 G > A).

**Conclusion:**

1. This study provides robust data supporting the application of whole-exome sequencing of family lines in clinical practice, offering valuable reference information for clinicians. 2. The newly discovered variants significantly enhance the relevant genetic databases. 3. Genetic diagnosis can offer clear guidance regarding the decision to continue with the pregnancy and future reproductive choices.

## 1 Introduction

Prenatal diagnosis is a method that uses advanced technology to assess the health of the fetus *in utero*. In cases where ultrasound examination indicates fetal structural abnormalities, prenatal diagnosis primarily includes non-invasive and invasive approaches. Non-invasive prenatal diagnosis utilizes imaging examinations and DNA technologies to evaluate fetal conditions without invasion. In contrast, invasive prenatal diagnosis uses direct sampling methods to obtain more precise genetic information about the fetus.

Ultrasound, as a routine screening method during pregnancy, can detect whether the fetus has significant structural abnormalities, ranging from single-system diseases to multi-system disorders. However, due to limitations in fetal phenotypes *in utero*, a diagnosis that relies solely on ultrasound examination often proves challenging. Therefore, supplementary diagnostic methods such as chromosome karyotype analysis, chromosome microarray analysis (CMA), and fluorescence *in situ* hybridization (FISH) are necessary. Whole-exome sequencing (WES) plays a crucial role in addressing issues involving single-nucleotide variants (SNVs). Its clinical application enables the detection of genetic variations in single or a few nucleotides during the fetal period, thereby enhancing the precision of prenatal diagnosis. Genetic diagnosis not only significantly impacts the prognosis of the fetus but also provides scientific bases for parental decisions and clinical management during pregnancy. Moreover, it offers guidance for both parents’ next pregnancy.

Currently, the majority of prenatal testing technologies focus on analyzing large genomic variations. Whole-exome sequencing (WES), as a novel detection technology based on high-throughput capture sequencing and deep sequencing, can accurately identify single-nucleotide variants (SNVs). It primarily targets the exons and flanking regions of approximately 20,000 coding genes in the human genome (1, 2). Although these regions constitute less than 2% of the human genome, over 85% of known pathogenic mutations originate from them (3). Compared to whole-genome sequencing (WGS), WES is more cost-effective (1), making it advantageous in clinical applications.

Several extensive prospective studies have confirmed the significant role of WES in prenatal diagnosis, particularly in cases where ultrasound examination indicates fetal structural abnormalities. WES can significantly enhance the genetic positive diagnosis rate, approximately 9% (4, 5). Moreover, small-scale studies demonstrate the high diagnostic capability and clinical utility of WES (6), providing insights into the etiology of recurrent miscarriages (7) and improving the detection rate of pathogenic variants in fetuses with congenital renal and urinary tract abnormalities (8). Sun et al. evaluated the likelihood of genetic abnormalities, particularly base mutations, in fetuses with congenital heart disease and coarctation of the aorta, identifying diagnostic genetic variants in 29% of cases through WES (82). This study highlights an overall WES diagnostic rate of 17% (5/30) within the cohort, underscoring the significant genetic role of WES. In prenatal diagnosis, excluding non-triploid and copy number variations, the use of WES to evaluate a group of fetuses with structural abnormalities and their parents has proven valuable (9). Mone et al. further expanded the sample size, confirming the diagnostic value of WES in fetuses with structural abnormalities (10).

Simultaneously, WES also holds potential for discovering new pathogenic genes, crucial for expanding the genetic spectrum. For instance, Tang et al. identified a novel variant *EYA1*: NM_000503.4:c.827-1G > C (intron 8, splicing mutation) associated with BOR in Chinese fetal families through WES, providing new avenues for early diagnosis of fetal diseases and prognosis guidance (83). In 2022, Lai et al. revealed the practicality of WES in the prenatal setting, recommending its widespread use beyond traditional examinations.

In recent years, fetal structural abnormalities’ detection rate has been approximately 2–4%. While traditional chromosomal analysis combined with CMA technology can identify genetic causes in 20% of these cases, the introduction of WES for genetic cause detection in majority of the unidentified cases provides robust support (11, 12). Many studies have indicated that WES often demonstrates the highest detection rates in multi-system abnormalities (11, 12), followed by skeletal dysplasia and central nervous system abnormalities (13). It also demonstrates exceptional diagnostic capabilities for the urinary tract abnormalities (14) and the cardiovascular system abnormalities (4, 5, 11, 12, 14, 15).

This study aims to delve into common pathogenic genes discoverable through WES in Jiangxi Province, aiming to improve the detection rate of genetic causes in fetal prenatal conditions, thereby reducing the birth rate of fetuses with congenital disabilities, and improving their prognosis.

## 2 Materials and methods

### 2.1 The participants of the study

From 25 February 2021 to 28 February 2023, a total of 357 cases of fetal ultrasound structural abnormalities underwent familial trio whole-exome sequencing following detailed genetic counseling and informed consent.

#### 2.1.1 Study design and participants

**Figure 1:**
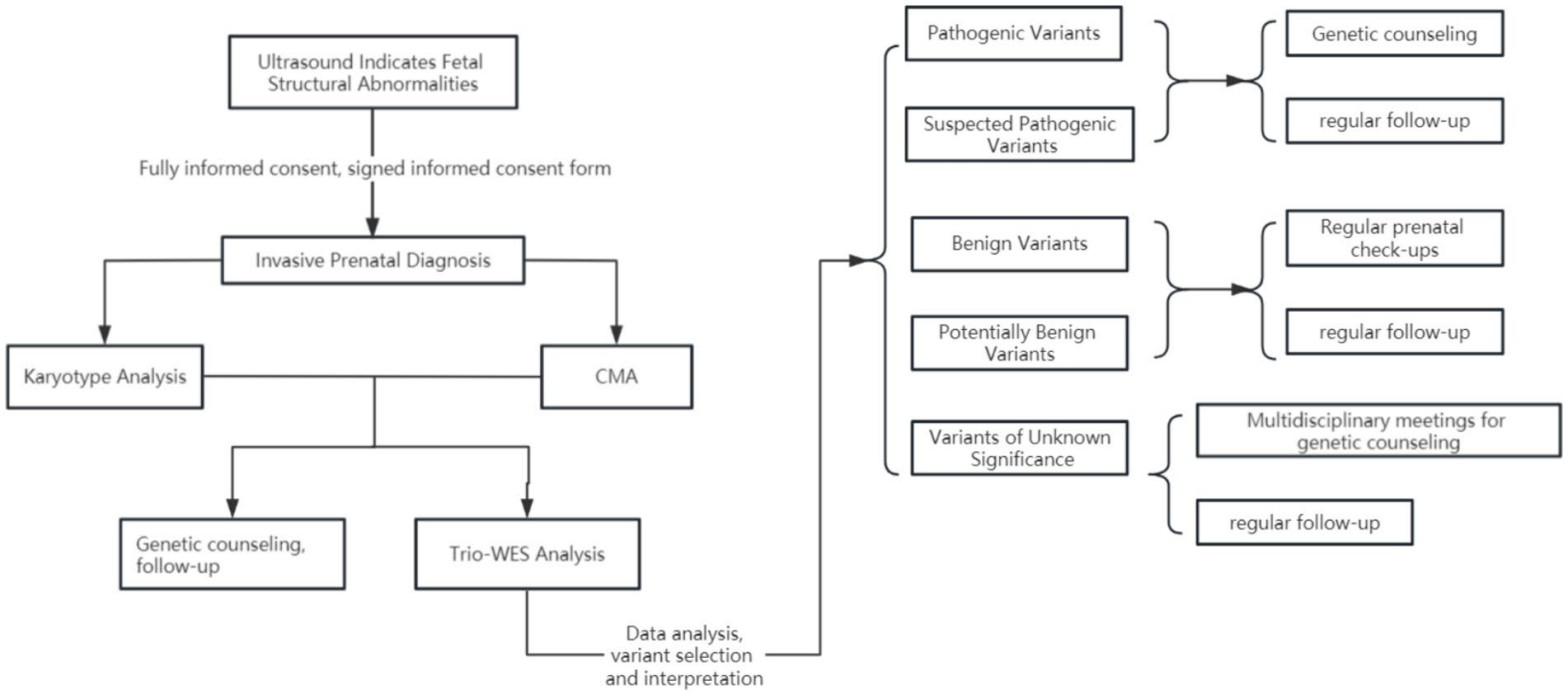
Overall experimental process diagram.

**Figure 2:**
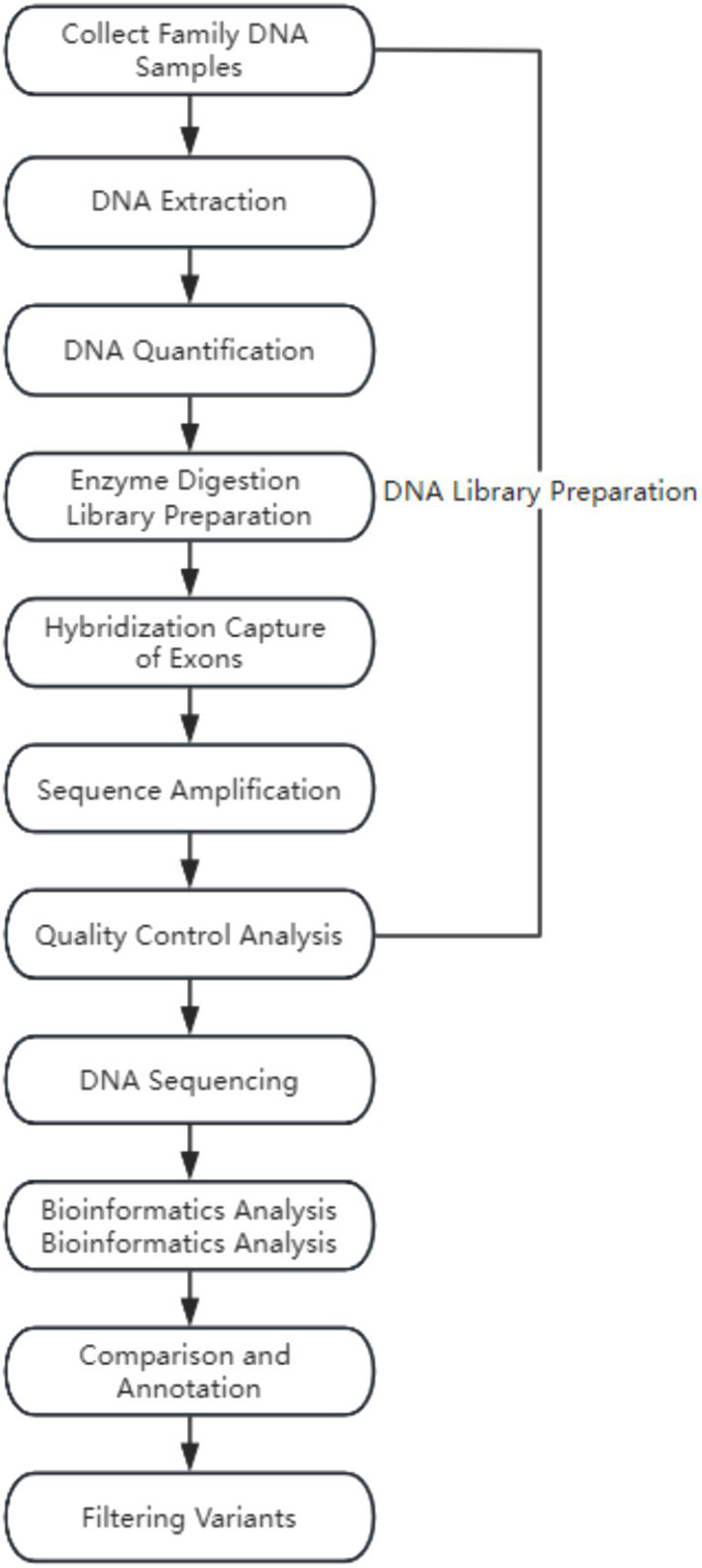
Whole-exome sequencing workflow diagram.

A total of 7,627 pregnant women presented at our hospital due to fetal structural abnormalities identified through systematic ultrasound examinations. Following the exclusion of cases in which chromosomal karyotyping and chromosome microarray analysis could ascertain the cause, as well as families that declined to participate in familial trio whole-exome sequencing (WES), 357 pregnant women provided their consent to undergo familial trio whole-exome sequencing. This included four cases in which chromosome microarray analysis identified potentially pathogenic segments; however, the analysis of familial trio WES determined that these segments lacked clinical significance (Figures 1, 2). The Clinical Research Ethics Committees of Jiangxi Maternal and Child Health Hospital approved this study.

#### 2.1.2 Inclusion and exclusion criteria

When fetal ultrasound examination indicates structural abnormalities, prenatal diagnostic physicians should provide detailed genetic counseling to the pregnant woman and her family to fully inform them about the risks of invasive prenatal diagnostic techniques and the purposes and limitations of various genetic testing methods, as well as to obtain signed informed consent. Common fetal structural abnormalities include multiple malformations, issues across various systems, hydrops fetalis, and several abnormal ultrasound soft markers. After excluding surgical contraindications, families that refused family-exome sequencing, and families with identified genetic pathogenic factors in chromosomal karyotype analysis and chromosomal microarray analysis (CMA), a total of 357 cases were included in the scope of the study (16–18).

### 2.2 Chromosomal karyotype analysis and CMA

Following comprehensive preoperative examinations, including temperature, pulse, heart rate, hematological tests, and ultrasound, pregnant women confirmed to have no surgical contraindications undergo the collection of chorionic villus, amniotic fluid, or umbilical cord blood samples. This collection process is strictly guided by real-time ultrasound examination to ensure accuracy and safety, with sample volumes of approximately 5–10 mL for chorionic villus tissue, 30 mL for amniotic fluid, or 3 mL for umbilical cord blood. After excluding maternal contamination, samples are promptly sent to the laboratory for detailed testing.

Chromosomal karyotype analysis requires the collection of chorionic villus, amniotic tissue, or cord blood samples, which are placed in specialized culture containers for routine *in vitro* cell culture processes. Subsequently, following fixation, glass slide preparation, baking, and banding analysis are conducted, typically involving counting 25 complete metaphase chromosomes to ensure accuracy in results. Next, analysis using the Leica chromosome automatic analyzer from Germany focuses on an in-depth examination of 5-well-dispersed karyotypes. If mosaicisms are detected during analysis, the number of counted karyotypes may be increased to 50–100, and mosaic ratios are calculated accordingly. Ultimately, chromosomal karyotype descriptions strictly adhere to the International System for Human Cytogenetic Nomenclature (ISCN2009) standards.

For CMA, genomic DNA from chorionic villus, amniotic fluid, or umbilical cord blood is first extracted following Qiagen kit procedures. Subsequently, high-throughput sequencing analysis is performed using the Affymetrix Cytoscan 750 chip from the American Affymetrix company, Thermo Fisher Scientific 901859. The interpretation of detected copy number variations follows the 2019 guidelines of the American College of Medical Genetics and Genomics (ACMG) for chromosomal copy number variations.

### 2.3 Whole-exome sequencing technology

#### 2.3.1 Detection methods

Whole-exome sequencing (WES) was performed on the genomic DNA of three individuals in a pedigree to identify variants associated with clinical phenotypes. Genomic DNA was isolated from the peripheral blood of both parents and fetal cord blood (amniotic fluid, chorionic villus) using the QIAamp DNA Mini Kit, QLAGEN 56304 (Qiagen). Subsequently, the isolated genomic DNA was randomly fragmented using the Covaris S220 ultrasonic processor, Covaris S220. Exome sequencing libraries were constructed following the manufacturer’s instructions and amplified on the high-throughput sequencing platform (MGISEQ-2000, BGI Tech, Beijing Genomics Institute) by fragment enrichment of target exons and adjacent cleavage sites. Finally, stringent quality control measures were applied to the raw sequencing data, such as removing adapter sequences and filtering low-quality sequences.

#### 2.3.2 Data analysis

The sequenced fragments were aligned to the UCSC GRCh37/hg19 human reference genome using the Burrows–Wheeler Aligner (BWA) software package, followed by removing duplicates and recalibrating base quality scores. Subsequently, the GATK HaplotypeCaller (Call germline SNPs and indels via local reassembly of haplotypes) was used for base quality score recalibration, including detecting single-nucleotide polymorphisms (SNPs) and insertions or deletions (indels). Finally, the ExomeDepth method (ExomeDepth is a R package designed to detect inherited copy number variants (CNVs) using high throughput DNA sequence data) was employed for copy number variation detection at the exon level.

#### 2.3.3 Variant filtering and interpretation

Genes were named according to the standards of the Human Genome Organization Gene Nomenclature Committee (HGNC), and variants were named following the guidelines of the Human Genome Variation Society (HGVS) (19). During variant annotation and filtering (20), clinical presentations of the proband, clinical presentations of both parents, population databases, disease databases, and bioinformatics prediction tools were integrated for variant selection. Finally, variants were classified for pathogenicity according to the guidelines of the American College of Medical Genetics and Genomics (ACMG) and the Association for Molecular Pathology (AMP). Detailed interpretation of pathogenic variants was referenced from the ClinGen Sequence Variant Interpretation Working Group and clinical literature reports.

#### 2.3.4 Clinical analysis of pedigree whole-exome sequencing results

In interpreting the experimental report, we adopted a comprehensive assessment strategy. First, we thoroughly correlated the detected variants in patients with relevant disease phenotypes, integrating ultrasound phenotypes, magnetic resonance imaging (MRI) examinations, and clinical presentations of both parents to make a comprehensive judgment. Additionally, multidisciplinary consultations were conducted with experts from various fields to assess the patient’s prognosis, provide professional opinions, and offer recommendations. This approach helps couples understand the fetal health status more accurately and provides valuable reference for future pregnancies.

### 2.4 Statistical analysis

In this study, we summarized and counted the genetic detection rates, pathogenic gene names, inheritance patterns, clinical presentations of associated diseases, relevant research reports, gene variant classifications, and ratings across various systems using whole-exome sequencing technology. Different types of data were represented using different methods. Count data were expressed as counts, percentages, or rates.

## 3 Experimental data analysis

### 3.1 Chromosome karyotype analysis, CMA, and WES examination results

The cohort recruited a total of 7,627 cases who presented at our hospital between 25 February 2021, and 28 February 2023, due to ultrasound-detected fetal structural abnormalities. Informed consent was obtained from all 7,627 families for karyotype and CMA testing. The study identified 848 cases (11.12%) with chromosomal abnormalities and 688 cases (9.02%) detected by CMA abnormalities, including 196 cases with concurrent chromosomal abnormalities. Among families willing to proceed with whole-exome sequencing (WES) after excluding chromosomal karyotype and CMA abnormalities, 357 cases were eligible. Ultimately, 33 cases (9.24% of 357) were confirmed according to study criteria. Table 1 summarizes the results of karyotype analysis, CMA, and WES testing for the 357 cases of ultrasound-detected fetal structural abnormalities.

**Table 1:** Karyotype analysis, CMA, and WES testing results for 357 cases.

Group	Number of tests	Variants detected related to phenotype
Karyotype	7627/7627	848 (848/7627, 11.12%)
CMA	7627/7627	688 (688/7627, 9.02%)
trio-WES	357/7627	33 (33/357, 9.24%)

### 3.2 WES detection rates by system

Among 357 fetuses, genetic etiology was definitively established using pedigree whole-exome sequencing combined with ultrasound phenotyping in 33 cases (9.24%). Among these, skeletal abnormalities were identified in 15 cases (45.45%), multiple malformations in 7 cases (21.21%), renal abnormalities in 3 cases (9.09%), multiple digital anomalies in 2 cases (6.06%) each, neurological abnormalities in 2 cases (6.06%) each, cleft lip/palate in 1 case (3.03%), cardiac abnormalities in 1 case (3.03%), hydrops fetalis in 1 case (3.03%), and cataract in 1 case (3.03%).

Among the 33 cases, 18 (54.54%) had *de novo* mutations, 8 (24.24%) inherited mutations from their fathers, 4 (12.12%) inherited mutations from their mothers, 1 (3.03%) had a homozygous mutation, and 2 (6.06%) had compound heterozygous mutations.

**Figure 3:**
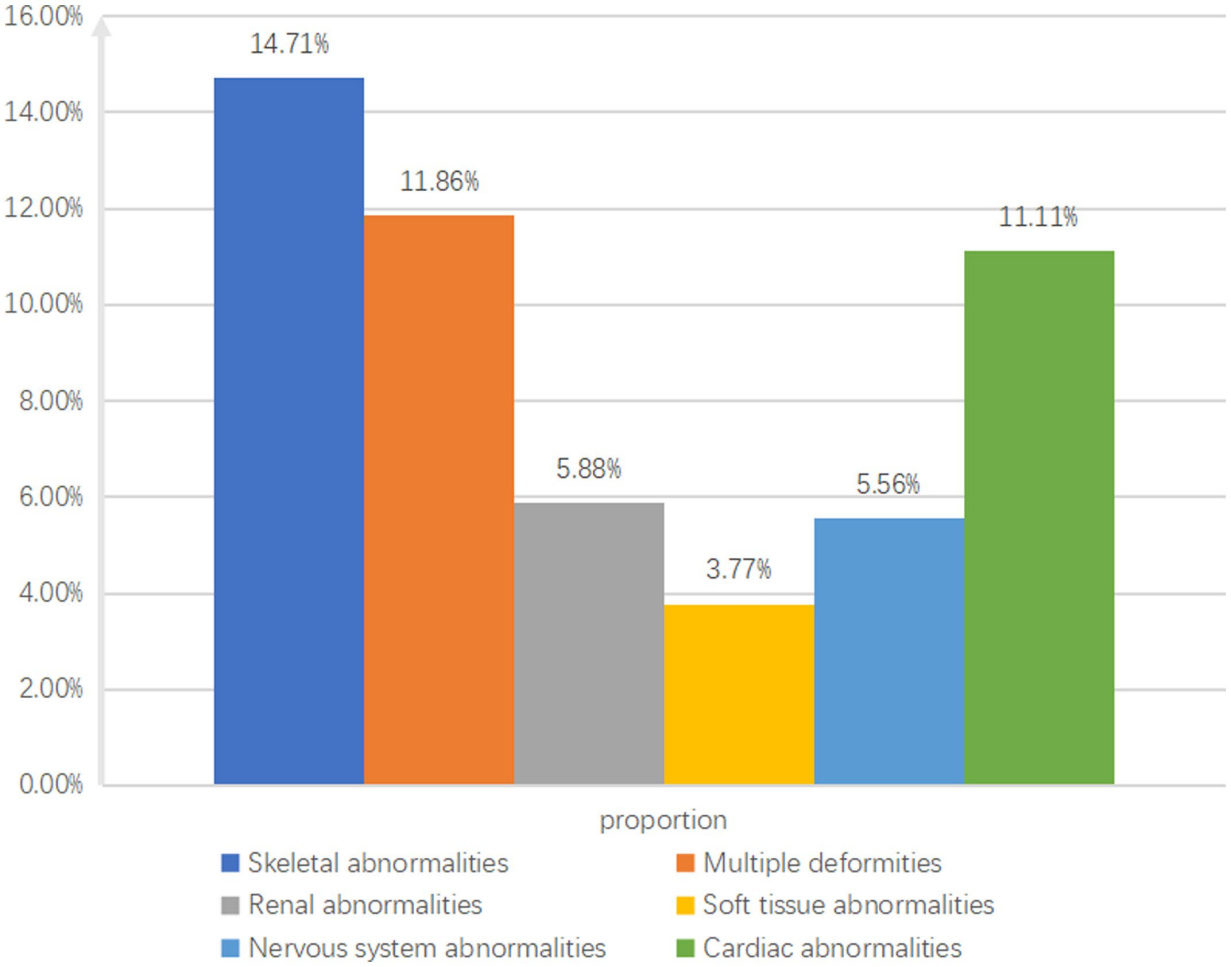
Classification of ultrasound abnormal indices.

The study identified 102 cases with skeletal system abnormalities, with pathogenic variants in 15 cases (14.71%); 59 cases with multiple malformations, with pathogenic variants in 7 cases (11.86%); 9 cases with cardiac abnormalities, with pathogenic variants in 1 case (11.11%); 51 cases with urogenital system abnormalities, with pathogenic variants in 3 cases (5.88%); 36 cases with neurological abnormalities, with pathogenic variants in 2 cases (5.56%); and 65 cases with multiple digital anomalies, with pathogenic variants in 2 cases (3.77%) (Figure 3).

### 3.3 Confirmed case genetic analysis

In cases of abnormal skeletal system, 5 cases are inherited from parents, and 10 cases are newly mutated. In case 15, the mutation is from the fetal father, who currently shows no clinical manifestations. Reports are confirming this mutation as pathogenic (21, 22), causing early-onset osteoporosis susceptibility type (OMIM:615221). *Disease characteristics:* Early-onset osteoporosis susceptibility type disease is often challenging to detect in early stages; it only draws attention when complications, such as frequent fractures (such as vertebral and rib fractures after minor trauma), low bone turnover markers, and reduced trabecular bone and cortical bone are observed. Age is a key factor influencing disease onset, particularly noticeable postmenopause in females and after 65 years of age in males. It is recommended for the male partner to maintain health regularly, inform both parents of the test results, and indicate that this mutation increases susceptibility to osteoporosis but does not directly cause disease. Clinical presentation of the disease has little relation to fetal ultrasound phenotype, and no clear pathogenic cause has been found. In case 11, the mutation is from the fetal father, who exhibits clubfoot. Reports are confirming this mutation as pathogenic (23, 24), causing Weyers Acrofacial Dysostosis (OMIM:193530). *Disease characteristics:* Weyers acrofacial dysostosis is a rare syndrome of ectodermal dysplasia with skeletal development abnormalities. The disease is sporadic, with an unknown prevalence, found only in a few families worldwide. In case 8, the mutation is from the fetal mother, who has a skeletal system disease. Reports are confirming this mutation as pathogenic (25–28). In case 9, the mutation is from the fetal father, who has short limbs. Reports are confirming this mutation as pathogenic (25, 29), causing Boomerang dysplasia (OMIM:112310). This disease affects overall skeletal development, with specific prevalence details unknown. In case 10, prenatal ultrasound examination revealed fetal limb shortening, with no reported family history of genetic disorders. Whole-exome sequencing identified two variants in the *EVC* gene (24). The first variant, NM_153717.2:c.130delC (p.Leu44Phefs*72), was inherited from the father who is also an asymptomatic carrier. This variant has not been previously reported in the literature as pathogenic. The second variant, NM_153717.2:EX9-EX11 Del, was inherited from the mother, who is also an asymptomatic carrier. These two compound heterozygous variants in *EVC* are associated with Weyers Acrofacial Dysostosis (OMIM:225500). *Disease characteristics:* Main clinical symptoms include chondrodystrophy, polydactyly (syndactyly), ectodermal dysplasia triad, and heart malformations. Patients also present with achondroplasia, short ribs, postaxial polydactyly, and defective nail and tooth development. 60% of achondrogenesis type-II patients also exhibit congenital heart defects, with the most common being an atrioventricular septal defect. Cases 1–7 have reports confirming this mutation as pathogenic (30–33), causing lethal osteogenesis imperfecta type I (OMIM:187600). This disease is a severe neonatal skeletal dysplasia where patients exhibit severe shortening of limbs with considerable head deformity, narrow chest, short ribs, and curved femurs. Some patients also show femoral fractures and bifid skull (30–33). Case 14 has reports confirming this mutation as pathogenic (34), causing limb-girdle short stature with small head syndrome—micrognathia and delayed development (OMIM:617164). *Disease characteristics:* Patients often present with limb-girdle short stature accompanied by micrognathia and/or micrognathia, where facial deformities, severe enteritis, microcephaly, limb-girdle short stature, and mild developmental delay characterize micrognathia. Case 12 has reports confirming this mutation as pathogenic (35), causing De Lange Syndrome Type 1 (OMIM:122470). *Disease characteristics:* It is a clinically heterogeneous developmental disorder affecting multiple systems, presenting with upper limb deformities, congenital heart disease, gastrointestinal malformations, and diaphragmatic hernia. In case 13, this mutation can cause King–Denborough syndrome (OMIM:619542). *Disease characteristics:* It is a congenital myopathy that easily leads to malignant hyperthermia, skeletal abnormalities, and facial deformities. The exact etiology is not fully understood, but some cases are associated with the ryanodine receptor gene.

A total of seven cases of multiple systemic abnormalities have reports confirming two cases inherited from parents, and five cases are *de novo* mutations. Case 16 is a mutation from the fetal mother; the mother has no clinical manifestations, and the reports confirm this mutation as pathogenic (36–40), causing glucose-6-phosphate dehydrogenase deficiency (OMIM:300908). This disease can cause chronic hemolytic anemia triggered by drugs, food, or infections. The five different categories of mutations are as follows: Type-I mutation, G6PD deficiency has a prevalence of less than 1/1,000,000; type-II mutation, G6PD enzyme activity is 1–10% of normal levels, associated with acute hemolytic anemia; type-III mutation, G6PD enzyme activity is 10–60% of normal levels; type-IV mutation, mild damage to G6PD enzyme activity, 60–100% of normal levels; and type-V mutation, increased G6PD enzyme activity. Fetal ultrasound phenotype does not correlate with clinical manifestations of the disease, no definite pathogenic variant found, informing both parents of test results, informing that fetal mutations from mothers are incomplete dominant inheritance, mothers have no symptoms suggesting good prognosis for fetuses but not ruling out the possibility of fetal onset, strict dietary control and careful drug selection are required after birth, while minimizing infection risks. In case 17, the mutation from the fetal father, the father is short statured, with irregular heartbeat, and the reports confirm this mutation as pathogenic (41), causing geleophysic dysplasia type 2 (OMIM:614185). *Disease characteristics:* A rare skeletal disorder characterized by short stature, significant hand and foot abnormalities, and distinctive facial features. Less than 30 cases have been reported. Other clinical features include progressive cardiac valve thickening, contractures of the Achilles tendon and heel, tracheal stenosis, bronchopulmonary insufficiency, and hepatomegaly. Fetal ultrasound phenotype shows minimal correlation with clinical manifestations of the disease, considering no clear pathogenic cause has been found; regular prenatal check-ups are recommended. Case 18 has reports confirming this mutation as pathogenic (42), causing tuberous sclerosis type 1 (OMIM:191100). The disease mainly affects organs such as the brain, kidneys, heart, and skin. Its main features include hamartomas (common benign proliferation of tissue or cells in an organ) and dysmorphic tissues (abnormal tissue binding). Case 19, a *de novo* mutation, causing craniosynostosis with clavicular skeletal dysplasia (OMIM:168550). *Disease characteristics:* Associated with cranial defects, characterized by enlarged cranial vault and clavicular ossification defect. Case 20 has reports confirming this mutation as pathogenic, causing spinal muscular atrophy type 2 (OMIM:253550) (43–45). *Disease characteristics:* SMA type II is a neuromuscular disorder characterized by degeneration of spinal anterior horn cells, leading to symmetric muscle weakness and atrophy. The disease is generally of moderate severity, with onset between 6–18 months. Patients are unable to stand and do not survive to adulthood, often associated with multi-system abnormalities. Case 21 has reports confirming this mutation as pathogenic (46), causing Noonan syndrome type 5 (OMIM:611553). *Disease characteristics:* Patients often present with short stature and varying degrees of facial deformities, with an increased risk of cardiac abnormalities. Fetal ultrasound phenotype does not correlate with clinical manifestations of the disease, no related pathogenic mutation found, informing both parents of test results and poor prognosis for Noonan syndrome type 5. Case 22 has reports confirming this mutation as pathogenic (47–49), causing Apert Syndrome (OMIM:101200). *Disease characteristics:* Newborn incidence is 1/65,000–1/88,000. Premature closure of cranial sutures is a typical symptom, inhibiting normal skull growth, affecting head and facial shape. Other symptoms include protruding and bulging eyes, shallow eye sockets, strabismus, and an underdeveloped upper jaw. The pathogenic gene for this disease is *FGFR2*, primarily involving missense mutations. Surgical intervention can prevent closure of cranial sutures to avoid brain damage.

Three cases of urinary tract abnormalities, two cases inherited from parents, and one case is a *de novo* mutation. Case 23 has a mutation from the fetal father, self-reports no clinical manifestations, advised abdominal ultrasound examination, which suggests bilateral kidney enlargement, and multiple cyst formation consistent with polycystic kidney disease. Reports confirm this mutation as pathogenic (50). Case 24, a *de novo* mutation, has reports confirming this mutation as pathogenic (51), and both can cause polycystic kidney disease type 1 (OMIM:173900), with about 50% of patients developing end-stage kidney disease around the age of 60. Fetal ultrasound phenotype corresponds to clinical manifestations of the disease, informing both parents of test results, disease risks, and considering the fetal mutation gene from the father. Although the father shows mild clinical signs, early detection of fetal kidney abnormalities is advised, with regular kidney function tests after birth, considering a generally fair prognosis for the fetus. Case 25, a mutation from the fetal mother, no clinical manifestations, has reports confirming this mutation as pathogenic (52–54), causing Emery–Dreifuss muscular dystrophy type 2 (OMIM:181350). This disease is a degenerative muscle disorder with a prevalence of 1:100,000. It can cause jaw and limb development abnormalities associated with type-A lipodystrophy (OMIM:248370), characterized mainly by underdeveloped jaw and clavicle, along with joint, soft tissue, and fat distribution abnormalities. It can also cause lethal restrictive dermopathy (OMIM:275210), characterized by fetal intrauterine growth restriction, skin erosions, and typically resulting in death within a week after birth. Both parents were informed of test results, fetal mutation gene from the mother, no corresponding clinical manifestations in the mother, considering autosomal recessive genetic disease, the fetus is a carrier with low disease probability. Ultrasound examination suggests bilateral kidney enlargement, without finding related pathogenic genes, recommending continued observation.

One case of cardiovascular system abnormality, case 26, has reports confirming this mutation as pathogenic (33, 55), causing Apert Syndrome (OMIM:101200). *Disease characteristics:* Newborn incidence is 1/65,000–1/88,000. Premature closure of cranial sutures is a typical symptom, inhibiting normal skull growth, affecting head and facial shape. Other symptoms include large and bulging eyes, shallow eye sockets, strabismus, an underdeveloped upper jaw, and lesions in important organs like the heart.

Two cases of nervous system abnormalities. Case 27 has reports confirming this mutation as pathogenic (56–58), causing Nijmegen Breakage Syndrome type 1 (OMIM:163950). This disease is characterized by short stature and varying facial deformities, with an increased risk of cardiac abnormalities. Informing both parents of test results after one month, and rechecking the ultrasound, fetal limbs are small with mild left ventricular reflux, consistent with clinical manifestations of the disease. Additionally, agenesis of the corpus callosum suggests poor prognosis, recommending termination of pregnancy. In case 28, this mutation can cause antenatal phenotypic abnormality (OMIM:NA). Characteristics include difficulties in weight gain and challenging nasogastric tube feeding, with early barriers in language and psychomotor development. Clinical manifestations of the disease often differ postnatally from the ultrasound phenotype, without finding related pathogenic mutations. Still, the *KMT2A* mutation is pathogenic, leading to more severe disease phenotypes, along with agenesis of the corpus callosum and poor prognosis.

Two cases of multiple soft marker abnormalities. Case 29 has reports confirming this mutation as pathogenic (47–49), causing Antley–Bixler syndrome without reproductive or steroid synthesis abnormalities (OMIM:207410). Features include craniosynostosis beginning in the perinatal period, midface hypoplasia, narrow or obstructed posterior nasal openings, bowed femora, and multiple joint contractures. Spider finger-like toes and/or congenital finger flexion have also been reported. Case 30, mutation from the fetal father, the father currently shows no clinical manifestations, advised abdominal ultrasound examination. Ultrasound examination indicates bilateral kidney enlargement; reports confirm that this mutation is pathogenic (51), causing polycystic kidney disease type 1 (OMIM:173900). Autosomal dominant polycystic kidney disease is a late-onset disease, typically manifesting in adulthood. Clinical manifestations include cysts in both kidneys and other organs such as the liver, pancreas, seminal vesicles, and arachnoid membrane, along with vascular abnormalities including intracranial aneurysms, aortic root dilation, aortic dissection, mitral valve prolapse, and abdominal hernia. Clinical manifestations in patients include hypertension, pain, and renal insufficiency. About 50% of patients develop end-stage kidney disease around 60 years of age. Fetal ultrasound phenotype does not match the clinical manifestations of the disease, without finding related pathogenic mutations.

Edematous fetus, case 31, mutation from fetal mother, no related disease manifestations in the mother, reports confirm this mutation as pathogenic (59), causing X-linked chondrodysplasia punctata type 1 (OMIM:302950). Characteristics include genetic spotted chondrodysplasia affecting skin and hair. Fetal ultrasound phenotype shows minimal correlation with clinical manifestations of the disease, without finding related pathogenic mutations and informing both parents of test results and of fetal generalized edema suggesting poor prognosis, recommending termination of pregnancy.

Cataract in case 32, a mutation from the fetal father, father diagnosed with cataracts, has reports confirming this mutation as pathogenic (60–62), causing cataract 4 (OMIM:115700). Characteristics include cataract, a common ophthalmological disease characterized by lens opacity, significantly affecting vision, potentially leading to blindness.

Cleft lip and palate in case 33, reports confirm this mutation as pathogenic (63–65), a mutation from the fetal father. Father shows skin deformities and hearing impairment, reports confirm this mutation as pathogenic (66, 67), causing Bart–Pumphrey syndrome (OMIM:149200). Characteristics include nail and skin deformities and hearing loss. Typical features include leukonychia, thick and brittle nails, and wart-like skin growths (acrochordons) at finger and toe joints. Patients often have palmoplantar keratoderma, severe to profound hearing loss in infancy, and skin deformities in childhood. Some patients may have no clinical manifestations. This disease is rare, and specific incidence rates are unclear. Fetal ultrasound phenotype does not correlate with clinical manifestations of the disease, without finding related pathogenic mutations.

Pregnancy outcomes included termination in 25 cases (25/33, 75.76%); premature birth in 3 cases (3/33, 9.09%), with 2 cases being healthy and 1 case presenting with a congenital clubfoot; cesarean section in 1 case (1/33, 3.03%), with the fetus diagnosed with trisomy 21; and full-term delivery in 4 cases (4/33, 12.12%), with 3 cases being healthy and 1 case showing fetal limb shortening (Table 2).

**Table 2:** Combined analysis of ultrasound phenotype with whole-exome sequencing results and pregnancy outcomes.

System/disease	Number	Ultrasound phenotype	Gene	cDNA alteration, protein alteration	ACMG classification	Clinical diagnosis	Genotype	Variant type	Pregnancy outcome
Skeletal system	1	Macrocephaly with short stature of the lower limbs	FGFR3	c.1138G > A (p.Gly380Arg)	PS3 + PS4 + PM1 + PM2 + PM6_Strong+PP1 + PP4	Thanatophoric dysplasia type 1/AD	*De novo* heterozygous	Missense	Artificial abortion
	2	Abnormal skeletal development of limbs, shortening, bending deformation, with a narrow chest cavity	FGFR3	c.742C > T (p.Arg248Cys)	PS2_Very Strong+PS3 + PS4 + PM2 + PP4	Thanatophoric dysplasia type 1/AD	*De novo* heterozygous	Missense	Artificial abortion
	3	Shortening of the humerus and femur	FGFR3	c.1138G > A (p.Gly380Arg)	PS3 + PS4 + PM1 + PM2 + PM6_Strong+PP1 + PP4	Lethal skeletal dysplasia type II/AD	*De novo* heterozygous	Missense	Artificial abortion
	4	Fetal biparietal diameter is large by 4 weeks, femur is small by 4 weeks	FGFR3	c.1138G > A (p.Gly380Arg)	PS3 + PS4 + PM1 + PM2 + PM6_Strong+PP1 + PP4	Thanatophoric dysplasia type 1/AD	*De novo* heterozygous	Missense	Artificial abortion
	5	Long bones of the limbs are short by 4 weeks	FGFR3	c.1138G > A (p.Gly380Arg)	PS3 + PS4 + PM1 + PM2 + PM6_Strong+PP1 + PP4	Thanatophoric dysplasia type 1/AD	*De novo* heterozygous	Missense	Artificial abortion
	6	Limbs are short by 6 weeks, considered chondrodysplasia	FGFR3	c.1138G > A (p.Gly380Arg)	PS3 + PS4 + PM1 + PM2 + PM6_Strong+PP1 + PP4	Thanatophoric dysplasia type 1/AD	*De novo* heterozygous	Missense	Artificial abortion
	7	Biparietal diameter > 95th percentile, femur < 1st percentile, polyhydramnios	FGFR3	c.1620C > A (p.Asn540Lys)	PS2 + PS4 + PM1 + PM2 + PP4	Thanatophoric dysplasia type 1/AD Crouzon syndrome—Cutaneous skeletal/AD	*De novo* heterozygous	Missense	self-reported normal fetus
	8	Multiple joint contractures in the feet, clubfoot, family history of genetic disease	FLNB	c.5071G > A (p.Gly1691Ser)	PM1 + PM2 + PP2 + PP3 + PP4 + PS4-Moderate+PM6_Strong	Boomerang dysplasia/AD	Maternal heterozygous	Missense	Artificial abortion
	9	Limbs short by three weeks, bright spot in left ventricle	FLNB	c.1945C > T (p.Arg649*)	PVS1 + PM2 + PM3_Strong	Boomerang dysplasia/AD	Paternal heterozygous	Duplication	Full-term birth, female, 7.1 pounds, short limbs
	10	Short limb development	EVC	c.130delC (p.Leu44Phefs*72) EX9-EX11 Del	PVS1 + PM2 + PP3 PVS1 + PM2 + PM3	Weyers acrofacial Dysostosis/AD Ehlers–Danlos Syndrome/AR	Paternal heterozygous Maternal heterozygous	Deletion Deletion	Artificial abortion
	11	Left congenital clubfoot varus	EVC2	c.2476C > T (p.Arg826*)	PVS1 + PM2 + PM3	Weyers acrofacial dysostosis/AD Antenatal phenotypic abnormality/AD	Paternal heterozygous	Nonsense	Premature male infant, 7 pounds, left congenital clubfoot varus
	12	Bilateral upper limb abnormalities at 14 weeks	NIPBL	c.5440C > T (p.Arg1814*)	PVS1 + PM2 + PP4	Cornelia de Lange syndrome type 1/AD	*De novo* heterozygous	Nonsense	Artificial abortion
	13	Abdominal circumference, femur, and humerus below fifth percentile, right superior vena cava with left branch	RYR1	c.14129 + 1G > A	PVS1 + PS2_Supporting+PM2	King–Denborough syndrome/AD Malignant hyperthermia susceptibility type 1/AD	*De novo* heterozygous	Missense	Artificial abortion
	14	Short mandible, retrognathia, limbs not matching clinical gestational age	ARCN1	c.934C > T (p.Arg312*)	PVS1 + PS2_Moderate+PM2	Limb-girdle short stature with minor head and mandibular dysplasia and developmental delay/AD	*De novo* heterozygous	Nonsense	Artificial abortion
	15	Femoral and humeral lengths less than the first percentile, right subclavian artery, vagus	WNT1	c.506G > A (p.Gly169Asp)	PM1 + PM2 + PM3_Strong+PP3 + PP4	Early-onset osteoporosis susceptibility/AD	Paternal heterozygous	Missense	Premature female infant, 3.7 pounds, healthy
Multiple systemic abnormalities	16	Thickened nuchal fold 6.2 mm, fetal tricuspid valve mild regurgitation	G6PD	c.1376G > T (p.Arg459Leu)	PS3 + PS4 + PM1 + PP4 + BS1	Glucose-6-phosphate dehydrogenase deficiency/XL	Maternal hemizygous	Missense	Cesarean section, one child, 6.4 pounds, with favism
	17	Umbilical cord edge echogenicity absent, possible umbilical cord cyst or placental cyst, premature ventricular contractions	FBN1	c.1090C > T (p.Arg364*)	PVS1 + PS4_Moderate+PM2_Supporting+PP4	Geleophysic dysplasia type 2/AD Acromicric dysplasia/AD	Paternal heterozygous	Duplication	Vaginal delivery, female infant, 5.8 pounds
	18	Bilateral periventricular nodular sclerosis with cardiac rhabdomyoma, ventricular	TSC1	c.737 + 1G > T	PVS1 + PS2 + PM2 + PP4	Tuberous sclerosis type 1/AD Antenatal phenotypic abnormality/AD	*De novo* heterozygous	Splice site	Artificial abortion
	19	Fetal occipital low echoic mass, possible meningocele, narrow transparent septum space	MSX2	c.423_427dupCAATC (p.Arg143Profs*39)	PVS1_Strong+PS2 + PM2	Foramen magnum with clavicular cranial dysplasia/AD Foramen magnum enlargement syndrome type 1/AD	*De novo* heterozygous	Duplication	Artificial abortion
	20	Multiple malformations	SMN1	EX7 Del	PVS1 + PM3 + PP4	Spinal muscular atrophy type 2/AR	Homozygous	deletion	Artificial abortion
	21	Possible overlapping fingers, fetal hydronephrosis, polyhydramnios	RAF1	c.1082G > C (p.Gly361Ala)	PS2_Very Strong+PM2 + PP2 + PP3	Noonan syndrome type 5/AD	*De novo* heterozygous	Missense	Artificial abortion
	22	Bilateral cleft hands with syndactyly, partial finger loss, bilateral toe syndactyly, enlarged posterior fossa	FGFR2	c.755C > G (p.Ser252Trp)	PS2 + PS3 + PS4_Supporting+PM1 + PM2 + PP2 + PP3 + PP4	Apert syndrome/AD Antley–Bixler syndrome without genital or steroid synthesis abnormalities/AD	*De novo* heterozygous	Missense	Artificial abortion
Urinary system	23	Increased renal parenchymal echoes bilaterally	polycystic kidney disease 1 (PKD1)	c.2054_2055delAG (p.Glu685Valfs*28)	PVS1 + PM2 + PP4	Autosomal dominant polycystic kidney disease type 1/AD	Paternal heterozygous	Deletion	Artificial abortion
	24	Multicystic kidneys	PKD1	c.3067C > T (p.Gln1023*)	PVS1 + PM2 + PP4	Autosomal dominant polycystic kidney disease type 1	Paternal heterozygous	Nonsense	Artificial abortion
	25	Bilateral renal enlargement, increased echogenicity	LMNA	c.1579C > T (p.Arg527Cys)	PVS1 + PM2 + PP4	Emery–Dreifuss muscular dystrophy type 2/AD Mandibular dysplasia with A-RLIPO/AR	Maternal heterozygous	Missense	Premature baby weighing 6 pounds, healthy
Cardiovascular system	26	Enlarged lateral ventricles, ventricular septal defect	FGFR2	c.758C > G (p.Pro253Arg)	PS2 + PM1 + PM2 + PP2 + PP4	Apert syndrome/AD	*De novo* heterozygous	Missense	Artificial abortion
Nervous system	27	Right lateral ventricular enlargement, left lateral ventricle at upper limit of normal, agenesis of corpus callosum, polyhydramnios	PTPN11	c.922A > G (p.Asn308Asp)	PS2_Very Strong+PS4 + PM1 + PM2 + PP1_Strong+PP2	Nijmegen breakage syndrome type 1/AD Multiple Lentigines syndrome type 1/AD Chondrosarcoma Syndrome/AD	*De novo* heterozygous	Missense	Artificial abortion
	28	Agenesis of corpus callosum, right lateral ventricle approximately 10.5-mm wide, no septum pellucidum	KMT2A	c.5332delA (p.Arg1778Glyfs*45)	PVS1 + PS2_Moderate+PM2	Antenatal phenotypic abnormality/AD Hirsutism, short stature, facial dysmorphism, and developmental delay/AD	*De novo* heterozygous	Deletion	Artificial abortion
Soft marker abnormalities	29	Multiple soft marker abnormalities	FGFR2	c.755C > G (p.Ser252Trp)	PS2 + PS3 + PS4_Supporting+PM1 + PM2 + PP2 + PP3 + PP4	Antley–Bixler syndrome without genital or steroid synthesis abnormalities/AD Apert syndrome/AD	*De novo* heterozygous	Missense	Artificial abortion
	30	Bilateral ventriculomegaly	PKD1	c.7111delG (p.Val2371Cysfs*11)	PVS1 + PM2 + PP4	Autosomal dominant polycystic kidney disease type 1/AD	Paternal heterozygous	Deletion	Full-term birth, female, 6 pounds, healthy
Edematous fetus	31	Generalized edema	ARSE	EX1-EX11E Del	PVS1 + PM2 + PS4-Support	X-linked chondrodysplasia punctata type 1/XL	Maternal hemizygous	Deletion	Artificial abortion
Cataract	32	Bilateral lens with complete strong echogenic manifestations	CRYGD	c.418C > T (p.Arg140*)	PVS1 + PS3 + PS4_Supporting+PM2 + PM6 + PP4	Congenital cataract type 4/AD	Paternal heterozygous	Nonsense	Artificial abortion
Cleft lip and palate	33	Cleft lip and palate	GJ1B2	c.235delC (p.Leu79Cysfs*3)	PVS1 + PS3_Moderate+PM3_Very Strong+BS1 PVS1 + PS3_Moderate+PM3_Very Strong	Bart–Pumphrey syndrome/AD Autosomal dominant deafness type 3A/AD Palmoplantar keratoderma with deafness/AD	*De novo* heterozygous Paternal heterozygous	Deletion Deletion	Artificial abortion

DM: disease-causing mutation; M: the mother is the patient; F: the father is the patient; /: n‌either the mother nor the father is a patient.

## 4 Discussion

With the advancement of ultrasound technology, more structural abnormalities in fetuses can be detected prenatally, with a detection rate of approximately 65.9%. This includes soft ultrasound marker abnormalities. Currently, whole-exome sequencing (WES) is not recommended for fetuses with isolated soft ultrasound marker abnormalities. In clinical practice, increased nuchal translucency positively correlates with fetal cardiac structural abnormalities and chromosomal abnormalities detection rates. Additionally, combined with advanced maternal age, high-risk maternal serum screening, or non-invasive DNA testing suggesting high risk, increased attention should be paid.

It is worth noting that, unlike our approach, Yates et al. performed whole-exome sequencing on fetuses who died due to ultrasound abnormalities (68). In 84 cases, they identified the genetic mechanism in 17 cases (20%). This suggests the importance of whole-exome sequencing in providing a precise diagnosis for fetuses that did not die due to termination of pregnancy or natural fetal death. Similarly, the study by Quinlan-Jones et al. (69) showed that combining exome sequencing with autopsy significantly increased the genetic diagnostic rate of structural abnormalities leading to termination of pregnancy, stillbirth, neonatal death, or infant mortality. Additionally, Best et al. compiled data from 31 prenatal whole-exome sequencing studies, revealing a rise in the genetic diagnosis rate of fetal abnormalities from 6.2 to 80%. This result once again highlights the potential of whole-exome sequencing technology for prenatal diagnosis (70).

During consultations for fetuses with isolated soft ultrasound marker abnormalities, the risks and limitations of current testing technologies should be fully disclosed, and both parents should be advised to undergo regular ultrasound examination observations.

Five patients ultimately gave birth to healthy infants. Case 25 involved phenotype assessment of both parents, indicating autosomal recessive inheritance in the fetus, who is a carrier without disease manifestation. Case 15 involved late-onset diseases without current clinical manifestations; there is an increased likelihood of osteoporosis due to multiple *WNT1* gene variants, which necessitates regular follow-up. Case 17 involved the *FBN1* gene, where the hidden inheritance pattern of this gene can explain the absence of relevant ultrasound phenotypes in the fetus. Case 30 considered adult-onset polycystic kidney disease, with symptoms typically appearing after the age of 40, making early detection challenging. Regular observation is recommended for patients. In case 7, the mother declined to mention the current status of the fetus during follow-up, claiming normalcy without undergoing neonatal examination. The reliability of these results is uncertain. Furthermore, the *FGFR3* gene also exhibits the possibility of autosomal dominant inheritance. However, combined with prenatal ultrasound examination indicating macrosomia and brachydactyly type 1, we still suspect fetal disease.

Research indicates that WES has the highest diagnostic rates for skeletal abnormalities and multiple malformations, at 14.71 and 11.86%, respectively, which are lower compared to previous studies (4, 5). This discrepancy may arise from various factors, including differences in ultrasound technology’s technician expertise and reporting scope. In this study, soft ultrasound marker abnormalities were classified separately. When combined with other systemic abnormalities or multiple abnormalities, WES is recommended. Patients with isolated soft ultrasound marker abnormalities may experience underdiagnosis. Common skeletal abnormality-related genes include *FGFR3*, *COL1A1*, *COL1A2*, and *DYNC2H1*. *FGFR3* is the most commonly identified in this study, likely due to its distinct clinical phenotype. Ultrasound examination is more sensitive to detecting fetal limb shortening and growth retardation. This is partly due to clear diagnostic criteria for fetal limb shortening and lower technical requirements for detecting limb abnormalities than cardiac abnormalities, resulting in higher clinical detection rates.

FGFRs are a family of fibroblast growth factor receptors, typical receptor tyrosine kinases involved in embryonic development, angiogenesis, and cartilage formation, regulating cartilage growth. *COL1A1* encodes the pro-α1 chain of type I collagen, which forms fibrillar collagen critical for connective tissue, bone, cornea, dermis, and tendons. *COL1A2* encodes the α2 chain, which, along with two α1 chains encoded by *COL1A1*, forms a triple helical structure stabilized by hydrogen bonds, maintaining its stability. Mutations in *COL1A1* and *COL1A2* genes can lead to osteogenesis imperfecta and congenital osteoporosis (71).

The *PKD1* gene is located on the short arm of chromosome 16, specifically on band 16p13.1 (72). It is primarily associated with the pathogenesis of polycystic kidney disease (PKD). This study identified three cases related to this gene. Among them, two cases showed early clinical manifestations related to renal issues, while one case exhibited clinical ultrasound examination findings suggestive of bilateral ventriculomegaly, later confirmed with fetal renal abnormalities. Adult-onset PKD is more common, with symptoms appearing later and often not clinically evident. Variations inherited from parents are more frequent. The study found that both parents usually perceive themselves as normal, but an ultrasound examination can detect certain issues.

During clinical consultations for fetuses, it is crucial to advise carriers of this gene, either the father or mother, to undergo regular ultrasound examination observations to prevent severe complications. Patients (either parent) often find it challenging to accept the diagnosis of being a carrier, requiring clinical psychologists to guide them, emphasizing the controllability of the disease and the importance of regular follow-up observations.

The study identified three novel mutation sites, contributing to the expansion of the gene database. Case 19 discovered the mutation *MSX2*; NM_002449.4: c.423_427dupCAATC (p.Arg143Profs*39) through WES. Both parents were normal. Prenatal ultrasound examination indicated a hypoechoic mass in the occipital region of the fetus, suggesting possible meningocele, and a narrow transparent septum. *MSX2* has been reported to play a crucial role in forming cranial bone morphology (73). In 1993, Li et al. demonstrated through *in situ* hybridization experiments that *MAX2* transcripts are present in osteoblasts adjacent to cranial sutures in mice (74). Subsequently, Li et al. discovered a (CA)n polymorphism in the *MSX2* gene related to craniosynostosis in studies by Warman et al. (75) and Müller et al. (76), primarily affecting the seventh amino acid, where histidine replaces proline. Florisson et al. found this to be an autosomal dominant mutation. The newly discovered mutation site spans from the 143rd amino acid to the 181st amino acid, resulting in changes in the last six amino acids within the second domain, disrupting the interaction between *MAX2* and TFIIF components in osteoblasts, leading to dysregulation of the bone sialoprotein promoter activity (77). Comprehensive analysis indicates this site as pathogenic, corroborated by fetal ultrasound examination showing related neurological manifestations, further confirming the pathogenicity of this mutation.

Case 10: WES identified the mutation *EVC*; NM_153717.2: c.130delC (p.Leu44Phefs*72). Prenatal ultrasound examination indicated fetal limb shortening, and the mutation originated from the fetus’s father. The mechanism of disease caused by the *EVC* gene remains unclear. In 2000, Ruiz-Perez et al. detected a heterozygous mutation in the *EVC* gene in a patient with Weyers acrofacial dysostosis (78). This mutation affects the first structural domain. This case represents a compound heterozygous mutation, and the fetal ultrasound phenotype of shortened limbs aligns with phenotypes associated with the *EVC* gene.

Case 13: WES revealed the mutation *RYR1*; NM_000540.2: c.14129 + 1G > A, a novel mutation. Prenatal ultrasound examination indicated fetal measurements (abdominal circumference, femur, and humerus) below the fifth percentile, with a right superior vena cava seen in the left branch. In 1998, Manning et al. identified 21 *RYR1*-related mutations in families with malignant hyperthermia, including four associated with central core myopathy (79). In 2001, Brandt et al. found susceptibility to malignant hyperthermia associated with 30 mutations in the *RYR1* gene (80). In 2005, Monnier et al. reported that 60% of mutations in a cohort labeled “confirmed” were in the MH1 (52%) and MH2 (36%) domains of the *RYR1* gene (81). Database entries indicate that this locus is related to King–Denborough syndrome, which can cause abnormalities in the skeletal and muscular systems, dwarfism, and intellectual development impacts, consistent with the corresponding ultrasound phenotype. Both parents opted for termination.

The study also identified cases 15, 16, 17, 28, 30, 31, and 33, where fetuses with clinically inconspicuous but genetically diagnosed conditions were unexpectedly discovered. Such discoveries effectively prompt early prevention strategies, mitigate potential triggers for disease onset, and enable targeted treatments. However, the uncertainty of ultrasound phenotypes complicates clinical interpretations. During follow-up, it was noted that pathogenic genes exhibiting fetal manifestations are more likely to be accurately diagnosed and draw attention from both parents. In contrast, exact clinical diagnoses for terminated fetuses remain challenging.

The study has limitations. First, sequencing is limited to exons, lacking relevance for some intronic diseases. Second, diagnostic reliance on ultrasound examination findings of fetal structural abnormalities varies by sonographer skill, potentially missing cases with incomplete penetrance, subtle phenotypic abnormalities, or delayed-onset disorders like intellectual developmental delay. Third, due to the risks of invasive prenatal diagnosis, few families opt for whole-exome sequencing, introducing selection bias and affecting sample size in systematic reviews.

## 5 Conclusion

Family-based WES plays a genetic diagnostic role in fetuses with ultrasound structural abnormalities, increasing diagnostic rates by 9.24%.

1. With the continuous enrichment of gene and disease databases, family-based whole-exome sequencing will play a more significant role. At the same time, discoveries of new loci will also expand the gene pool.
2. Genetic diagnosis guides decisions on whether to continue pregnancies and affects the reproduction of the entire family.

Currently, the pathogenesis of some ultrasound structural abnormalities remains unclear. With technological advancements, comprehensive analyses combining whole genome sequencing, optical genome mapping, and other technologies assist in diagnosis.

As technology advances, the potential of whole genome research in clinical applications becomes increasingly prominent. More detailed genetic analyses and interpretations of numerous variations will be crucial directions for future research. Currently, clinical studies on whole genome sequencing in critically ill newborns have yielded a diagnostic positivity rate of 20% (3/15), providing a reliable genetic basis for clinical management of critically ill cases and demonstrating the immense value of WGS in disease diagnosis. In tuberculosis molecular epidemiology, whole genome sequencing has shown unique advantages, deeply exploring drug resistance in tuberculosis bacteria to provide substantial evidence for developing effective treatment strategies and playing a critical role in mixed infection diagnosis, helping doctors accurately assess patient conditions. Furthermore, the application of whole genome sequencing in prenatal diagnosis has achieved significant results, demonstrating its generalizability and substantial research value across various fields. With ongoing technological improvements and decreasing costs, whole genome sequencing is expected to see the broader application in the future and represents a promising area for in-depth research.

## Data Availability

The datasets presented in this study can be found in online repositories. This data can be found here: 10.6084/m9.figshare.29396441.

## References

[1] Miceikaite I Fagerberg C Brasch-Andersen C Torring PM Kristiansen BS Hao Q. Comprehensive prenatal diagnostics: Exome versus genome sequencing. Prenat Diagn. (2023) 43:1132–41. doi: 10.1002/pd.6402, PMID: 37355983

[2] Yaldiz B Kucuk E Hampstead J Hofste T Pfundt R Corominas Galbany J. Twist exome capture allows for lower average sequence coverage in clinical exome sequencing. Hum Genomics. (2023) 17:39. doi: 10.1186/s40246-023-00485-5, PMID: 37138343 PMC10155375

[3] van Dijk EL Auger H Jaszczyszyn Y Thermes C. Ten years of next-generation sequencing technology. Trends Genet. (2014) 30:418–26. doi: 10.1016/j.tig.2014.07.001, PMID: 25108476

[4] Lord J McMullan DJ Eberhardt RY Rinck G Hamilton SJ Quinlan-Jones E. Prenatal exome sequencing analysis in fetal structural anomalies detected by ultrasonography (PAGE): a cohort study. Lancet. (2019) 393:747–57. doi: 10.1016/S0140-6736(18)31940-8, PMID: 30712880 PMC6386638

[5] Petrovski S Aggarwal V Giordano JL Stosic M Wou K Bier L. Whole-exome sequencing in the evaluation of fetal structural anomalies: a prospective cohort study. Lancet. (2019) 393:758–67. doi: 10.1016/S0140-6736(18)32042-7, PMID: 30712878

[6] Vilarinho S Mistry PK. Exome sequencing in clinical Hepatology. Hepatology. (2019) 70:2185–92. doi: 10.1002/hep.30826, PMID: 31222768 PMC6885087

[7] Pangalos C Hagnefelt B Lilakos K Konialis C. First applications of a targeted exome sequencing approach in fetuses with ultrasound abnormalities reveals an important fraction of cases with associated gene defects. Peer J. (2016) 4:e 1955. doi: 10.7717/peerj.1955 PMC4860337 27168972

[8] Lei TY Fu F Li R Wang D Wang RY Jing XY. Whole-exome sequencing for prenatal diagnosis of fetuses with congenital anomalies of the kidney and urinary tract. Nephrol Dial Transplant. (2017) 32:1665–75. doi: 10.1093/ndt/gfx031, PMID: 28387813

[9] He M Du L Xie H Zhang L Gu Y Lei T. The added value of whole-exome sequencing for anomalous fetuses with detailed prenatal ultrasound and postnatal phenotype. Front Genet. (2021) 12:627204. doi: 10.3389/fgene.2021.627204, PMID: 34367232 PMC8340955

[10] Mone F Abu Subieh H Doyle S Hamilton S Mcmullan DJ Allen S. Evolving fetal phenotypes and clinical impact of progressive prenatal exome sequencing pathways: cohort study. Ultrasound Obstet Gynecol. (2022) 59:723–30. doi: 10.1002/uog.24842, PMID: 34940998

[11] Fu F Li R Yu Q Wang D Deng Q Li L. Application of exome sequencing for prenatal diagnosis of fetal structural anomalies: clinical experience and lessons learned from a cohort of 1618 fetuses. Genome Med. (2022) 14:123. doi: 10.1186/s13073-022-01130-x, PMID: 36307859 PMC9615232

[12] Lai T Au L Lau Y Lo HM Chan K Cheung KW. Application of prenatal whole exome sequencing for structural congenital anomalies-experience from a local prenatal diagnostic laboratory. Healthcare (Basel). (2022) 10:2521. doi: 10.3390/healthcare10122521, PMID: 36554045 PMC9778831

[13] Yaron Y Ofen Glassner V Mory A Zunz Henig N Kurolap A Bar Shira A. Exome sequencing as first-tier test for fetuses with severe central nervous system structural anomalies. Ultrasound Obstet Gynecol. (2022) 60:59–67. doi: 10.1002/uog.24885, PMID: 35229910 PMC9328397

[14] Lei TY Fu F Li R Yu QX Du K Zhang WW. Whole-exome sequencing in the evaluation of fetal congenital anomalies of the kidney and urinary tract detected by ultrasonography. Prenat Diagn. (2020) 40:1290–9. doi: 10.1002/pd.5737, PMID: 32436246

[15] Li R Fu F Yu Q Wang D Jing X Zhang Y. Prenatal exome sequencing in fetuses with congenital heart defects. Clin Genet. (2020) 98:215–30. doi: 10.1111/cge.13774, PMID: 32410215

[16] Diderich K Joosten M Govaerts L Van Opstal D Go A Knapen M. Is it feasible to select fetuses for prenatal WES based on the prenatal phenotype. Prenat Diagn. (2019) 39:1039–40. doi: 10.1002/pd.5522, PMID: 31506969 PMC6899996

[17] Gao C Wang X Mei S Li D Duan J Zhang P. Diagnostic yields of trio-WES accompanied by CNVseq for rare neurodevelopmental disorders. Front Genet. (2019) 10:485. doi: 10.3389/fgene.2019.00485, PMID: 31178897 PMC6542989

[18] Deden C Neveling K Zafeiropopoulou D Gilissen C Pfundt R Rinne T. Rapid whole exome sequencing in pregnancies to identify the underlying genetic cause in fetuses with congenital anomalies detected by ultrasound imaging. Prenat Diagn. (2020) 40:972–83. doi: 10.1002/pd.5717, PMID: 32333414 PMC7497059

[19] Richards S Aziz N Bale S Bick D Das S Gastier-Foster J. Standards and guidelines for the interpretation of sequence variants: a joint consensus recommendation of the American College of Medical Genetics and Genomics and the Association for Molecular Pathology. Genet Med. (2015) 17:405–24. doi: 10.1038/gim.2015.30, PMID: 25741868 PMC4544753

[20] Zastrow DB Baudet H Shen W Thomas A Si Y Weaver MA. Unique aspects of sequence variant interpretation for inborn errors of metabolism (IEM): the Clin gen IEM working group and the phenylalanine hydroxylase gene. Hum Mutat. (2018) 39:1569–80. doi: 10.1002/humu.23649, PMID: 30311390 PMC6556116

[21] Liu Y Song L Ma D Lv F Xu X Wang J. Genotype-phenotype analysis of a rare type of osteogenesis imperfecta in four Chinese families with WNT1 mutations. Clin Chim Acta. (2016) 461:172–80. doi: 10.1016/j.cca.2016.07.012, PMID: 27450065

[22] Li L Mao B Li S Xiao J Wang H Zhang J. Genotypic and phenotypic characterization of Chinese patients with osteogenesis imperfecta. Hum Mutat. (2019) 40:588–600. doi: 10.1002/humu.23718, PMID: 30715774

[23] Valencia M Lapunzina P Lim D Zannolli R Bartholdi D Wollnik B. Widening the mutation spectrum of EVC and EVC2: ectopic expression of Weyer variants in NIH 3T3 fibroblasts disrupts hedgehog signaling. Hum Mutat. (2009) 30:1667–75. doi: 10.1002/humu.21117, PMID: 19810119

[24] Nguyen TQ Saitoh M Trinh HT Doan NM Mizuno Y Seki M. Truncation and microdeletion of EVC/EVC2 with missense mutation of EFCAB7 in Ellis-van Creveld syndrome. Congenit Anom (Kyoto). (2016) 56:209–16. doi: 10.1111/cga.12155, PMID: 26748586

[25] Krakow D Robertson SP King LM Morgan T Sebald ET Bertolotto C. Mutations in the gene encoding filamin B disrupt vertebral segmentation, joint formation and skeletogenesis. Nat Genet. (2004) 36:405–10. doi: 10.1038/ng1319, PMID: 14991055

[26] Farrington-Rock C Firestein MH Bicknell LS Superti-Furga A Bacino CA Cormier-Daire V. Mutations in two regions of FLNB result in atelosteogenesis I and III. Hum Mutat. (2006) 27:705–10. doi: 10.1002/humu.20348, PMID: 16752402

[27] Zhang D Herring JA Swaney SS McClendon TB Gao X Browne RH. Mutations responsible for Larsen syndrome cluster in the FLNB protein. J Med Genet. (2006) 43:e24. doi: 10.1136/jmg.2005.038695, PMID: 16648377 PMC2564529

[28] Bicknell LS Farrington-Rock C Shafeghati Y Rump P Alanay Y Alembik Y. A molecular and clinical study of Larsen syndrome caused by mutations in FLNB. J Med Genet. (2007) 44:89–98. doi: 10.1136/jmg.2006.043687, PMID: 16801345 PMC2598053

[29] Farrington-Rock C Kirilova V Dillard-Telm L Borowsky AD Chalk S Rock MJ. Disruption of the Flnb gene in mice phenocopies the human disease spondylocarpotarsal synostosis syndrome. Hum Mol Genet. (2008) 17:631–41. doi: 10.1093/hmg/ddm188, PMID: 17635842 PMC2680151

[30] Placone J Hristova K. Direct assessment of the effect of the Gly 380Arg achondroplasia mutation on FGFR3 dimerization using quantitative imaging FRET. PLoS One. (2012) 7:e46678. doi: 10.1371/journal.pone.0046678, PMID: 23056398 PMC3467271

[31] Xue Y Sun A Mekikian PB Martin J Rimoin DL Lachman RS. FGFR3 mutation frequency in 324 cases from the international skeletal dysplasia registry. Mol Genet Genomic Med. (2014) 2:497–503. doi: 10.1002/mgg3.96, PMID: 25614871 PMC4303219

[32] Ajmal M Mir A Shoaib M Malik SA Nasir M. Identification and in silico characterization of p.G380R substitution in FGFR3, associated with achondroplasia in a non-consanguineous Pakistani family. Diagn Pathol. (2017) 12:47. doi: 10.1186/s13000-017-0642-3 PMC5499044 28679403

[33] Zhang J Li J Saucier JB Feng Y Jiang Y Sinson J. Non-invasive prenatal sequencing for multiple Mendelian monogenic disorders using circulating cell-free fetal DNA. Nat Med. (2019) 25:439–47. doi: 10.1038/s41591-018-0334-x, PMID: 30692697

[34] Janine R Stephan R Marianne G Anja S Daniel K Volker O. Transient N-glycosylation abnormalities likely due to a de novo loss-of-function mutation in the delta subunit of coat protein I. Am J Med Genet A. (2019) 179:1371–5. doi: 10.1002/ajmg.a.61190, PMID: 31075182

[35] Moog U Felbor U Has C Zirn B. Disorders caused by genetic mosaicism. Dtsch Arztebl Int. (2020) 116:119–25. doi: 10.3238/arztebl.2020.0119, PMID: 32181732 PMC7081367

[36] Li P Thompson JN Wang X Song L. Analysis of common mutations and associated haplotypes in Chinese patients with glucose-6-phosphate dehydrogenase deficiency. Biochem Mol Biol Int. (1998) 46:1135–43. doi: 10.1080/15216549800204692, PMID: 9891846

[37] Iwai K Hirono A Matsuoka H Kawamoto F Horie T Lin K. Distribution of glucose-6-phosphate dehydrogenase mutations in Southeast Asia. Hum Genet. (2001) 108:445–9. doi: 10.1007/s004390100527, PMID: 11499668

[38] Jiang W Yu G Liu P Geng Q Chen L Lin Q. Structure and function of glucose-6-phosphate dehydrogenase-deficient variants in Chinese population. Hum Genet. (2006) 119:463–78. doi: 10.1007/s00439-005-0126-5, PMID: 16607506

[39] Yan T Cai R Mo O Zhu D Ouyang H Huang L. Incidence and complete molecular characterization of glucose-6-phosphate dehydrogenase deficiency in the Guangxi Zhuang autonomous region of southern China: description of four novel mutations. Haematologica. (2006) 91:1321–8. PMID: 17018380

[40] Chen Y Xiu W Dong Y Wang J Zhao H Su Y. Mutation of glucose-6-phosphate dehydrogenase deficiency in Chinese Han children in eastern Fujian. Medicine (Baltimore). (2018) 97:e11553. doi: 10.1097/MD.0000000000011553, PMID: 30045279 PMC6078762

[41] Zhurayev R Proost D Zerbino D Fedorenko V Meester JA Laer VAN. Identification of FBN1 gene mutations in Ukrainian Marfan syndrome patients. Genet Res (Camb). (2016) 98:e13. doi: 10.1017/S0016672316000112, PMID: 27724990 PMC6865158

[42] Su Y Wei Q Wan J Li L. Tuberous sclerosis complex: early screening and infant outcome in NICU. J Trop Pediatr. (2021) 67:fmab 012 [pii]. doi: 10.1093/tropej/fmab012, PMID: 33693890

[43] Ogino S Wilson RB. Genetic testing and risk assessment for spinal muscular atrophy (SMA). Hum Genet. (2002) 111:477–500. doi: 10.1007/s00439-002-0828-x, PMID: 12436240

[44] Alías L Bernal S Fuentes-Prior P Barceló MJ Also E Martínez-Hernández R. Mutation update of spinal muscular atrophy in Spain: molecular characterization of 745 unrelated patients and identification of four novel mutations in the SMN1 gene. Hum Genet. (2009) 125:29–39. doi: 10.1007/s00439-008-0598-1, PMID: 19050931

[45] Butchbach M. Genomic variability in the survival motor neuron genes (SMN1 and SMN2): implications for spinal muscular atrophy phenotype and therapeutics development. Int J Mol Sci. (2021) 22:7896. doi: 10.3390/ijms22157896, PMID: 34360669 PMC8348669

[46] Harms FL Alawi M Amor DJ Tan TY Cuturilo G Lissewski C. The novel RAF1 mutation p.(Gly 361Ala) located outside the kinase domain of the CR3 region in two patients with Noonan syndrome, including one with a rare brain tumor. Am J Med Genet A. (2018) 176:470–6. doi: 10.1002/ajmg.a.38569, PMID: 29271604

[47] Chen P Zhang L Weng T Zhang S Sun S Chang M. A Ser 252Trp mutation in fibroblast growth factor receptor 2 (FGFR2) mimicking human Apert syndrome reveals an essential role for FGF signaling in the regulation of endochondral bone formation. PLoS One. (2014) 9:e87311. doi: 10.1371/journal.pone.0087311, PMID: 24489893 PMC3904987

[48] Kunwar F Tewari S Bakshi SR. Apert syndrome with S252W FGFR2 mutation and characterization using Phenomizer: an Indian case report. J Oral Biol Craniofac Res. (2017) 7:67–71. doi: 10.1016/j.jobcr.2016.07.002, PMID: 28316926 PMC5343159

[49] Dan H Huang X Xing Y Shen Y. Application of targeted panel sequencing and whole exome sequencing for 76 Chinese families with retinitis pigmentosa, Mol genet. Genomic Med. (2020) 8:e 1131. doi: 10.1002/mgg3.1131, PMID: 31960602 PMC7057118

[50] Rossetti S Chauveau D Kubly V Slezak JM Saggar-Malik AK Pei Y. Association of mutation position in polycystic kidney disease 1 (PKD1) gene and development of a vascular phenotype. Lancet. (2003) 361:2196–201. doi: 10.1016/S0140-6736(03)13773-7, PMID: 12842373

[51] Kim H Park HC Ryu H Kim H Lee HS Heo J. Genetic characteristics of Korean patients with autosomal dominant polycystic kidney disease by targeted exome sequencing. Sci Rep. (2019) 9:16952. doi: 10.1038/s41598-019-52474-1, PMID: 31740684 PMC6861305

[52] Agarwal AK Kazachkova I Ten S Garg A. Severe mandibuloacral dysplasia-associated lipodystrophy and progeria in a young girl with a novel homozygous Arg 527Cys LMNA mutation. J Clin Endocrinol Metab. (2008) 93:4617–23. doi: 10.1210/jc.2008-0123, PMID: 18796515 PMC2626450

[53] Luo DQ Wang XZ Meng Y He DY Chen YM Ke ZY. Mandibuloacral dysplasia type A-associated progeria caused by homozygous LMNA mutation in a family from southern China. BMC Pediatr. (2014) 14:256. doi: 10.1186/1471-2431-14-256, PMID: 25286833 PMC4287574

[54] Malashicheva A Bogdanova M Zabirnyk A Smolina N Ignatieva E Freilikhman O. Various Lamin a/C mutations alter expression profile of mesenchymal stem cells in mutation specific manner. Mol Genet Metab. (2015) 115:118–27. doi: 10.1016/j.ymgme.2015.04.006, PMID: 25982065

[55] Chen CP Su YN Chang TY Chern SR Chen CY Su JW. Rapid detection of de novo P253R mutation in FGFR2 using uncultured amniocytes in a pregnancy affected by polyhydramnios, Blake's pouch cyst, and Apert syndrome, Taiwan. J Obstet Gynecol. (2013) 52:273–7. doi: 10.1016/j.tjog.2013.04.022, PMID: 23915865

[56] Tartaglia M Kalidas K Shaw A Song X Musat DL van der Burgt I. PTPN11 mutations in Noonan syndrome: molecular spectrum, genotype-phenotype correlation, and phenotypic heterogeneity. Am J Hum Genet. (2002) 70:1555–63. doi: 10.1086/340847, PMID: 11992261 PMC379142

[57] Yamamoto GL Aguena M Gos M Hung C Pilch J Fahiminiya S. Rare variants in SOS2 and LZTR1 are associated with Noonan syndrome. J Med Genet. (2015) 52:413–21. doi: 10.1136/jmedgenet-2015-103018, PMID: 25795793

[58] Koh AL Tan ES Brett MS Lai A Jamuar SS Ng I. The spectrum of genetic variants and phenotypic features of southeast Asian patients with Noonan syndrome. Mol Genet Genomic Med. (2019) 7:e00581. doi: 10.1002/mgg3.581 30784236 PMC6465663

[59] Matos-Miranda C Nimmo G Williams B Tysoe C Owens M Bale S. A prospective study of brachytelephalangic chondrodysplasia punctata: identification of arylsulfatase E mutations, functional analysis of novel missense alleles, and determination of potential phenocopies. Genet Med. (2013) 15:650–7. doi: 10.1038/gim.2013.13, PMID: 23470839

[60] Vendra VP Agarwal G Chandani S Talla V Srinivasan N Balasubramanian D. Structural integrity of the Greek key motif in βγ-crystallins is vital for central eye lens transparency. PLoS One. (2013) 8:e70336. doi: 10.1371/journal.pone.0070336, PMID: 23936409 PMC3735602

[61] Zhai Y Li J Zhu Y Xia Y Wang W Yu Y. A nonsense mutation of γD-crystallin associated with congenital nuclear and posterior polar cataract in a Chinese family. Int J Med Sci. (2014) 11:158–63. doi: 10.7150/ijms.7567, PMID: 24465161 PMC3894400

[62] Jia H Ma Q Liang Y Wang D Chang Q Zhao B. Clinical and genetic characteristics of Chinese patients with congenital cranial dysinnervation disorders. Orphanet J Rare Dis. (2022) 17:431. doi: 10.1186/s13023-022-02582-5, PMID: 36494820 PMC9733177

[63] Choung YH Moon SK Park HJ. Functional study of GJB2 in hereditary hearing loss. Laryngoscope. (2002) 112:1667–71. doi: 10.1097/00005537-200209000-00026, PMID: 12352684

[64] Yan D Park HJ Ouyang XM Pandya A Doi K Erdenetungalag R. Evidence of a founder effect for the 235delC mutation of GJB2 (connexin 26) in east Asians. Hum Genet. (2003) 114:44-50. doi: 10.1007/s00439-003-1018-1, PMID: 14505035

[65] Duan S Guo Y Chen X Li Y. Genetic mutations in patients with nonsyndromic hearing impairment of minority and Han Chinese ethnicities in Qinghai. China, J Int Med Res. (2021) 49:3000605211000892. doi: 10.1177/03000605211000892, PMID: 33827324 PMC8040579

[66] Zhang Y Wang J Li L Sun Y Feng B. Three common GJB2 mutations causing nonsyndromic hearing loss in Chinese populations are retained in the endoplasmic reticulum. Acta Otolaryngol. (2010) 130:799–803. doi: 10.3109/00016480903443191 20095872

[67] Ji YB Han DY Lan L Wang DY Zong L Zhao FF. Molecular epidemiological analysis of mitochondrial DNA12SrRNA A1555G, GJB2, and SLC26A4 mutations in sporadic outpatients with nonsyndromic sensorineural hearing loss in China. Acta Otolaryngol. (2011) 131:124–9. doi: 10.3109/00016489.2010.483479, PMID: 21162657 PMC3528947

[68] Yates CL Monaghan KG Copenheaver D Retterer K Scuffins J Kucera CR. Whole-exome sequencing on deceased fetuses with ultrasound anomalies: expanding our knowledge of genetic disease during fetal development. Genet Med. (2017) 19:1171–8. doi: 10.1038/gim.2017.31, PMID: 28425981

[69] Quinlan-Jones E Lord J Williams D Hamilton S Marton T Eberhardt RY. Molecular autopsy by trio exome sequencing (ES) and postmortem examination in fetuses and neonates with prenatally identified structural anomalies. Genet Med. (2019) 21:1065–73. doi: 10.1038/s41436-018-0298-8, PMID: 30293990 PMC6752266

[70] Best S Wou K Vora N Van der Veyver IB Wapner R Chitty LS. Promises, pitfalls and practicalities of prenatal whole exome sequencing. Prenat Diagn. (2018) 38:10–9. doi: 10.1002/pd.5102, PMID: 28654730 PMC5745303

[71] Takegaki J Sase K Kono Y Nakano D Fujita T Konishi S. Intramuscular injection of mesenchymal stem cells activates anabolic and catabolic systems in mouse skeletal muscle. Sci Rep. (2021) 11:21224. doi: 10.1038/s41598-021-00627-6, PMID: 34707171 PMC8551189

[72] Baselet B Sonveaux P Baatout S Aerts A. Pathological effects of ionizing radiation: endothelial activation and dysfunction. Cell Mol Life Sci. (2019) 76:699–728. doi: 10.1007/s00018-018-2956-z, PMID: 30377700 PMC6514067

[73] Yapijakis C Pachis N Sotiriadou T Vaila C Michopoulou V Vassiliou S. Molecular mechanisms involved in Craniosynostosis. In Vivo. (2023) 37:36–46. doi: 10.21873/invivo.13052, PMID: 36593018 PMC9843758

[74] Jabs EW Müller U Li X Ma L Luo W Haworth IS. A mutation in the homeodomain of the human MSX2 gene in a family affected with autosomal dominant craniosynostosis. Cell. (1993) 75:443–50. doi: 10.1016/0092-8674(93)90379-5, PMID: 8106171

[75] Warman ML Mulliken JB Hayward PG Müller U. Newly recognized autosomal dominant disorder with craniosynostosis. Am J Med Genet. (1993) 46:444–9. doi: 10.1002/ajmg.1320460420 8357019

[76] Müller U Warman ML Mulliken JB Weber JL. Assignment of a gene locus involved in craniosynostosis to chromosome 5qter. Hum Mol Genet. (1993) 2:119–22. doi: 10.1093/hmg/2.2.119, PMID: 8499900

[77] Newberry EP Latifi T Battaile JT Towler DA. Structure-function analysis of Msx 2-mediated transcriptional suppression. Biochemistry. (1997) 36:10451–62. doi: 10.1021/bi971008x, PMID: 9265625

[78] Ruiz-Perez VL Ide SE Strom TM Lorenz B Wilson D Woods K. Mutations in a new gene in Ellis-van Creveld syndrome and Weyers acrodental dysostosis. Nat Genet. (2000) 24:283–6. doi: 10.1038/73508 10700184

[79] Manning BM Quane KA Ording H Urwyler A Tegazzin V Lehane M. Identification of novel mutations in the ryanodine-receptor gene (RYR1) in malignant hyperthermia: genotype-phenotype correlation. Am J Hum Genet. (1998) 62:599–609. doi: 10.1086/301748, PMID: 9497245 PMC1376943

[80] Brandt A Schleithoff L Jurkat-Rott K Klingler W Baur C Lehmann-Horn F. Screening of the ryanodine receptor gene in 105 malignant hyperthermia families: novel mutations and concordance with the in vitro contracture test. Hum Mol Genet. (1999) 8:2055–62. doi: 10.1093/hmg/8.11.2055, PMID: 10484775

[81] Monnier N Kozak-Ribbens G Krivosic-Horber R Nivoche Y Qi D Kraev N. Correlations between genotype and pharmacological, histological, functional, and clinical phenotypes in malignant hyperthermia susceptibility. Hum Mutat. (2005) 26:413–25. doi: 10.1002/humu.20231, PMID: 16163667

[82] Sun H. Han L. Hao X. Chen Z. Wang J. Yi T.. (2022). Genetic abnormalities in fetal congenital heart disease with aberrant right subclavian artery. Sci Rep 12, 15899. doi: 10.1038/s41598-022-20037-6 36151134 PMC9508080

[83] Tang P. Li J. Li J. Yang J. Zhu J. (2022). Prenatal diagnosis and genetic analysis of a fetus with Branchio-oto-renal syndrome: A case report. Medicine (Baltimore) 101, e31172. doi: 10.1097/MD.0000000000031172 36316881 PMC9622624

[84] Li H. Durbin R. (2010) Fast and accurate long-read alignment with Burrows-Wheeler Transform. Bioinformatics, Epub. 10.1093/bioinformatics/btp698 PMC2828108 20080505

